# Downstream of the HOX genes: Explaining conflicting tumour suppressor and oncogenic functions in cancer

**DOI:** 10.1002/ijc.33949

**Published:** 2022-02-15

**Authors:** Richard Morgan, Keith Hunter, Hardev S. Pandha

**Affiliations:** ^1^ School of Biomedical Sciences University of West London London UK; ^2^ Unit of Oral and Maxillofacial Pathology, School of Clinical Dentistry University of Sheffield Sheffield UK; ^3^ Faculty of Health and Medical Sciences University of Surrey Guildford UK

**Keywords:** HOX, oncogene, PBX, tumour suppressor

## Abstract

The HOX genes are a highly conserved group of transcription factors that have key roles in early development, but which are also highly expressed in most cancers. Many studies have found strong associative relationships between the expression of individual HOX genes in tumours and clinical parameters including survival. For the majority of HOX genes, high tumour expression levels seem to be associated with a worse outcome for patients, and in some cases, this has been shown to result from the activation of pro‐oncogenic genes and pathways. However, there are also many studies that indicate a tumour suppressor role for some HOX genes, sometimes with conclusions that contradict earlier work. In this review, we have attempted to clarify the role of HOX genes in cancer by focusing on their downstream targets as identified in studies that provide experimental evidence for their activation or repression. On this basis, the majority of HOX genes would appear to have a pro‐oncogenic function, with the notable exception of HOXD10, which acts exclusively as a tumour suppressor. HOX proteins regulate a wide range of target genes involved in metastasis, cell death, proliferation and angiogenesis, and activate key cell signalling pathways. Furthermore, for some functionally related targets, this regulation is achieved by a relatively small subgroup of HOX genes.

## Graphical abstract

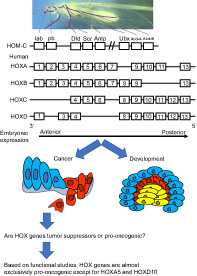

**Abbreviations**
DAPK1: death‐associated protein kinase 1
EGFR: epidermal growth factor receptor
EMT: epithelial to mesenchymal transition
ER: oestrogen receptor
HNSCC: head and neck squamous cell carcinoma
IGFBP‐3: insulin‐like growth factor binding protein‐3
ITGA2: gene encoding integrin A2
IκBα: inhibitor of NF‐κB alpha
lncRNA: long noncoding RNA
MEIS: myeloid ecotropic viral integration site 1 homolog
MMP: matrix metalloproteinase
NEP: neutral endopeptidase
PBX: pre‐B‐cell leukaemia homeobox
PDEF: prostate‐derived Ets factor
PD‐L2: programmed death protein ligand 2
PDPK1: 3‐phosphoinositide‐dependent protein kinase 1
RTK: receptor tyrosine kinase
SAMD9: sterile alpha motif domain‐containing protein 9
TDO2: tryptophan 2,3‐dioxygenase
VASP: vasodilator‐stimulated phosphoprotein
VEGF: vascular endothelial growth factor
VEGFR: vascular endothelial growth factor receptor

## 1 INTRODUCTION

The HOX genes are a family of transcription factors first characterised for their role in the early development of insects, and subsequently found to have close homologues in vertebrates with strikingly conserved functions, including the determination of cell and tissue identity.^1^, ^2^ In Drosophila, there is a single chromosomal cluster of HOX genes, the members of which are expressed along the anterior to posterior embryonic axis in the same order, both spatially and temporally, as they are in the cluster, with the most 3′ member expressed first and most anteriorly. This cluster has been duplicated twice in the course of evolution, such that mammals have four clusters, the members of which are also expressed in the same order along the anterior to posterior axis, with the 3′ member expressed first, reflecting a closely conserved regulation of HOX genes between invertebrates and vertebrates.^3^

In humans, the four clusters of HOX genes are named A, B, C and D, and are located on chromosomes 7, 17, 12 and 2, respectively. The individual genes within each cluster are numbered consecutively from the 3′ end, so, for example, the first gene in the HOXB complex is named HOXB1. The genes in the equivalent position in each of the other complexes are paralogous, having descended from the same ancestral HOX genes, thus HOXA4, HOXC4 and HOXD4 are paralogous of HOXB4. In general, proteins encoded by genes within the same paralogue group have similar sequences, and often have similar functions,^2^, ^3^ although a notable exception, HOXC10 and HOXD10, is described later in this review. A number of HOX genes have been lost during evolution, meaning that in total the four HOX clusters contain 39 genes.^3^ In mammals, HOX genes are expressed in overlapping patterns along the main anterior to posterior axis during development, with for example HOXB1 expressed in the developing hindbrain and HOXB9 in the posterior half of the spinal cord. Likewise, similar nested sequences of expression occur in other structures, including the limbs and many of the organs.^1^, ^2^, ^3^

HOX transcription factors contain a highly conserved DNA binding domain, the homeodomain that facilitates binding to a 4‐base pair sequence. Increased binding affinity and specificity is conferred by various co‐factors which are themselves transcription factors, including members of the pre‐B‐cell leukaemia homeobox (PBX) and myeloid ecotropic viral integration site 1 homolog (MEIS) families.^4^ This facilitates both activation and repression of gene transcription in a context‐dependent manner. In addition, HOX proteins have roles in the cytoplasm, although these are less well studied. A notable example is the binding of HOXA10 to p38 MAPK leading to its dephosphorylation and hence attenuation of p38 MAPK/STAT3 signalling.^5^ Thus, the cellular location of HOX proteins is important in determining their function, and is in turn influenced by posttranslational modifications that regulate cofactor binding.^6^ For example, acetylation of a lysine residue in HOXB9 (AcK27‐HOXB9) causes it to become localised to the cytoplasm, preventing it from transcriptionally regulating its target genes.^7^

In addition to their role in development, it is now well established that HOX genes are strongly expressed in most types of cancer, including prostate,^8^ breast,^9^ lung,^10^ renal,^11^ head and neck^12^ and ovarian^13^ cancer, as well as mesothelioma.^14^ Although the expression of certain HOX genes is often reported as being characteristic of specific cancers, for example, HOXC4 and HOXC6 in prostate cancer,^8^ generally the majority of the 39 HOX genes are significantly upregulated. There have been numerous studies on the role of HOX genes in cancer, which can be divided broadly into associative studies (establishing a statistically significant relationship between HOX gene expression and key clinical parameters such as disease‐free survival), and mechanistic studies, in which for example the downstream target genes of HOX proteins are identified with experimental evidence for a role in cancer development or progression, using molecular and cell biology approaches such as gene knock out, reduction of expression using short interfering RNAs, or gain of function experiments through the forced expression of a HOX gene. There are many more associate studies than mechanistic ones, although many mechanistic studies also include associative data.

To date, the overall picture that emerges from these studies is rather ambiguous—while some indicate that a particular HOX gene has a tumour suppressor function, others have ascribed it a pro‐oncogenic function in the same cancer. In this review, we have focused on mechanistic studies in which molecular and cellular evidence is provided to support a tumour suppressor vs oncogenic function for different HOX genes. On this basis, the majority of HOX genes actually have a pro‐oncogenic function. Our analysis reveals that the HOX genes activate (or more rarely repress) a wide range of genes involved in metastasis, cell death, proliferation and angiogenesis, and also activate key cell signalling pathways. While some of these functionally distinct groups of target genes are regulated by many members of the HOX family, others are activated by just a few paralogue groups. HOX genes reported to have both tumour suppressor and pro‐oncogenic activities might be context‐dependent with respect to cancer type and background gene expression.

## 2 ONCOGENIC vs TUMOUR SUPPRESSOR FUNCTIONS OF HOX GENES

It is now clear from the growing body of work across multiple cancer types that while the majority of HOX genes seem to be associated with oncogenesis, a significant minority instead have tumour suppressor functions. This has made it more difficult to come to a global understanding of HOX function in cancer, as for example, some HOX genes are reported to have oncogenic functions in around half of the published studies, but tumour suppressor functions in other studies. Excluding studies that are based only on associative studies (eg, the expression levels of a HOX gene compared to overall survival), and focusing instead on mechanistic studies where the function of the HOX gene in cancer is directly addressed, may help provide a clearer view (Table 1). First, based on mechanistic studies, it becomes apparent that the majority of HOX genes have an oncogenic function. Most HOX genes, including HOXB7,^15^, ^16^, ^17^, ^18^, ^19^, ^20^, ^21^, ^22^, ^23^, ^24^, ^25^, ^26^, ^27^, ^28^, ^29^, ^30^, ^31^, ^32^, ^33^, ^34^, ^35^, ^36^, ^37^, ^38^, ^39^, ^40^, ^41^, ^42^, ^43^, ^44^, ^45^, ^46^, ^47^ HOXB8^48^, ^49^, ^50^, ^51^, ^52^, ^53^, ^54^ and HOXC10^55^, ^56^, ^57^, ^58^, ^59^, ^60^, ^61^, ^62^, ^63^, ^64^, ^65^, ^66^, ^67^, ^68^, ^69^, ^70^, ^71^, ^72^ are only reported to have oncogenic functions, while only two, HOXA5^73^, ^74^, ^75^, ^76^, ^77^, ^78^, ^79^, ^80^, ^81^, ^82^, ^83^ and HOXB13,^84^, ^85^, ^86^, ^87^, ^88^, ^89^, ^90^, ^91^, ^92^, ^93^, ^94^, ^95^, ^96^, ^97^, ^98^, ^99^, ^100^, ^101^, ^102^, ^103^, ^104^, ^105^, ^106^, ^107^, ^108^ are notable for having oncogenic functions reported in around 50% of publications, and tumour suppressor functions in the other 50%. Only one HOX gene, HOXD10, seems to act almost exclusively as a tumour suppressor, albeit with a notable exception in head and neck squamous cell carcinoma (HNSCC).^109^, ^110^, ^111^, ^112^, ^113^, ^114^, ^115^, ^116^, ^117^, ^118^, ^119^, ^120^, ^121^

**TABLE 1:** HOX genes as tumour suppressors or oncogenes in different cancers

Cancer	Tumour suppressor function	Oncogenic function
Ovarian	A4	A10, A13, B4 (Akt/PI3K upregulated), B9 (increased expression of EMT markers such as vimentin, MMP9 and Oct4), B13 (HOXB13 collaborates with activated ras/direct binding to the Slug promoter), C10 (promotes EMT via increased Slug expression)
Hepatic cellular carcinoma	D10 (represses ERK signalling)	A5, C4 (activates Snail and TGF‐β signalling), C10 (upregulates metastasis‐related genes, including PDPK1 and VASP), D3 (HOXD3 binds to promoter region of ITGA2 and up‐regulates its expression, thus activating ERK1/2 signalling), D3 (HOXD3 can directly target the promoter region of VEGFR and increase VEGFR expression), D9 (HOXD9 can interact with the promoter region of ZEB1 and promotes ZEB1 expression—increased EMT)
Glioblastoma	A11, D10	A5, A9, B8 (activation of the PI3K/AKT pathway and expression of EMT‐related genes, possibly through direct binding to the promoter of SAMD9), B13 (direct transcriptional upregulation of lncRNA HOXC‐AS3), C6 (increases the phosphorylation of Jun amino‐terminal kinase, ERK and P38, as well as the expression of MAPK signalling‐related genes, including c‐myc, c‐jun and p53), C9 (suppresses Beclin1‐mediated autophagy by directly inhibiting the transcription of DAPK1), C10 (increases PI3K/AKT signalling)
Oesophageal	D10 (suppresses the activation of the PI3K/AKT/mTOR signalling pathway)	A5 (wnt/βcat), A13, B2 (activates NANOG and cMYC transcription), B5, B7, B13 (increased NF‐κB/p65 signalling), C6, C13 (direct repression of caspase 3)
Lung		A5, B2, B5 (increased wnt/β‐catenin signalling), B7 (TGF‐β/SMAD3, VEGFA, MMP2), B9 (increased MMP9 expression and wnt/β‐catenin signalling), B13 (increased ABCG1, EZH2 and Slug expression), C8 (upregulation of TGF‐β1, downregulation of E‐cadherin), C10 (enhanced phosphorylation of PI3K, increased expression of MMP2/9, VCAM‐1, vimentin and E‐cadherin; increased BET/MEK signalling), C13 (increased cyclin D1 and cyclin E1 expression—entry into G1), D3 (E‐cadherin expression lost and plakoglobin strongly repressed, integrin alpha3 and beta3 up‐regulated and N‐cadherin and integrin alpha4 newly expressed)
Colorectal	A5 (inhibits wnt/β‐catenin signalling), D10 (inhibits AKT/MAPK signalling)	A6, B5 (elevated CXCR4 and integrin beta expression), B7 (increased DNA repair factor KU70/80 expression), B8 (increased wnt/β‐catenin signalling, increased expression of MMP2, c‐Myc, CyclinD1, vimentin; STAT3, Vimentin, N‐cadherin, Twist, Zeb1 and Zeb2, downregulation of E‐cadherin), B9 (increased angptl2, CXCL1, IL8 and TGF‐β1 expression), B13 (increased NF‐κB/p65 signalling), C4, D3 (increased integrin β3 expression and MAPK/AKT signalling)
Breast	A5 (increased p53 and caspase 2 and 8 expression), A9, B4 (direct binding to StAR‐related lipid transfer domain protein 13), D10 (reduced Akt signalling)	A7 (increased ERα expression), B3, B5 (increased EGFR signalling), B7 (increased EGFR signalling, direct protein binding to ERα to increase HER2 expression; increased TGF‐β expression; direct binding and activation of EGFR promoter; loss of epithelial proteins, claudin 1 and claudin 7, mislocalization of claudin 4 and E‐cadherin and the expression of mesenchymal proteins vimentin and alpha‐smooth muscle actin), B9 (increased PI3K/AKT signalling; increased E2F1 expression), B13 (mediates tamoxifen resistance: suppresses ERα and induces IL‐6 expression activating the mTOR pathway via STAT3), C8 (increased cadherin‐11 expression), C10 (increased NF‐κB signalling, recruits homologous repair proteins), D3 (increased integrin αv and β3 expression, integrin β3‐mediated wnt/β‐catenin signalling)
Clear cell renal cell carcinoma	A6 (reduced PI3K/Akt signalling), A11 (reduced wnt signalling), D1 (reduced TGF‐β and wnt signalling)	
Pancreatic	D10 (reduced expression of survivin VEGF, MMP14 and N cadherin)	A10 (MMP3 expression via TGF‐β2‐mediated activation of the p38 MAPK pathway), B5 (increased GSK3β/β‐catenin signalling), B7 (increased ERK signalling; reduced BAX and BAD expression), B9
Bladder	D10 (increased E‐cadherin expression and reduced MMP14 expression)	A10 (increased FOSL1 and MMP3 expression)
Gastric	B9 (suppression of phosphorylated‐Akt and NF‐κB‐dependent Snail expression), D10 (direct transcriptional activation of IGFBP3 which in turn inhibits MMP14, uPA and uPAR; reduces RhoC‐AKT signalling)	A10 (increased JAK1/STAT3 signalling and BCL2 expression), A13 (downregulation of Erk1/2; downregulation of DHRS2 leading to increased MDM2 expression; increased wnt/β‐catenin signalling), B5 (induces invasion and migration through direct transcriptional upregulation of β‐catenin, as well as its downstream target genes cyclin D1 and c‐Myc), B7 (activates EMT through the Src‐FAK pathway; increased pAKT and pMAPKs levels; B7 knockdown downregulates pAkt and upregulates PTEN; increased PIK3R3/AKT signalling), B8 (increased EMT via elevated ZEB2 expression), C6 (increased MMP9 expression), C10 (increased ATM/NF‐κB and MAPK signalling; increased interleukin‐6, TNF‐α, TGF‐β and EGF expression), D9 (increased expression of RUFY3)
Prostate	A10, B13 (inhibition of AR by direct binding to TCF4 ‐TCF4 target genes cmyc and cyclin D1 also inhibited), D13 (reduced SMAD1 expression)	B3 (increased CDCA3 expression), B9 (increased PI3K/AKT signalling), B13 (increased CCL2/CCR2 cytokine and integrin signalling; increased MAPK signalling, increased ZnT zinc output transporter expression leading to reduced intracellular zinc concentrations and activation of NF‐κB‐mediated signalling), C4, C6 (increased NEP and IGFBP‐3 expression, direct regulation of BMP7, GFGR2, IGFBP3 and PDGFRA), C8 (increased expression of SRC‐3, a member of the SRC/p160 steroid receptor cofactor family)
Osteosarcoma	B1 (reduced NF‐kB signalling)	B7 (increased expression of MMP2, MMP7, p‐PI3K and p‐Akt), B8 (increased wnt/β‐catenin signalling), C8 (increased MMP9 expression), C10 (increased expression of Bcl‐2, MMP2 and MMP9, reduced expression of caspase 3 and E‐cadherin)
Head and neck squamous cell carcinoma		B5 (HOXB5 directly binds to the promoter region of EGFR and consequently increases Akt/wnt/β‐catenin signalling), C6 (increases BCL2 expression by direct promoter binding), D10

*Note*: HOX genes are listed if there is experimental evidence for them having either a tumour suppressor or oncogenic function in cancer. The key target genes and/or signalling pathways regulated by each HOX gene are shown in brackets if they have been clearly identified in the literature.

Abbreviations: DAPK1, death‐associated protein kinase 1; EMT, epithelial to mesenchymal transition; ER, oestrogen receptor; IGFBP‐3, insulin‐like growth factor binding protein‐3; ITGA2, gene encoding integrin A2; lncRNA, long noncoding RNA; PDPK1, 3‐phosphoinositide‐dependent protein kinase 1; NEP, neutral endopeptidase; SAMD9, sterile alpha motif domain‐containing protein 9; VASP, vasodilator‐stimulated phosphoprotein; VEGFR, vascular endothelial growth factor receptor.

How can apparently conflicting functions of genes such as HOXA5 and HOXB13 be explained? For the former, oncogenic vs tumour suppressor activity seems to be dependent only on cancer type, as it is reportedly oncogenic in glioma^75^ and oesophageal squamous cell cancer,^83^ but acts as a tumour suppressor in colorectal^78^ and breast cancer.^73^, ^81^ This might reflect the relative dependency of different signalling pathways in different cancers that are activated or repressed by downstream targets of HOXA5, although the wnt/β‐catenin pathway for example is activated by HOXA5 in oesophageal squamous cell cancer (in which it acts as an oncogene),^83^ but repressed by HOXA5 in colorectal cancer (in which it acts as a tumour suppressor),^78^ indicating that the explanation may be more complex. Further analysis is made difficult by the relative scarcity of mechanistic studies on HOXA5. However, considerably more has been published on HOXB13. In studies where HOXB13 is identified as promoting oncogenesis, it is frequently because it has a role in driving metastasis. In prostate cancer, this is through increasing the expression of integrin subunits (ITGAV and ITGB1) specific to prostate cancer bone metastasis,^96^ by preventing the expression of prostate‐derived Ets factor (PDEF) which inhibits metastasis,^90^ through blocking the transport of zinc ions which in turn increases NF‐κB‐mediated signalling by reducing inhibitor of NF‐κB alpha (IκBα) activity,^92^ and by promoting expression of the epithelial to mesenchymal transition (EMT)‐inducing Slug transcription factor.^105^ Conversely, tumour‐suppressor functions of HOXB13 in prostate cancer seem to depend on its ability to block cell division through the downregulation of cyclin D1 through competition for TCF4 binding to its promoter,^87^, ^88^ and androgen signalling through direct interaction with the androgen receptor.^89^, ^122^ Taken together, it would seem that high levels of HOXB13 expression in tumours may drive initial tumour growth but suppress subsequent metastasis. It should be noted though that there are several studies that conflict with this. For example, the G84E mutation in HOXB13 is thought to promote prostate cancer initiation, but not progression and metastasis.^123^ However, this mutation is thought to potentially weaken the interaction of HOXB13 with its MEIS cofactor, and the HOXB13/MEIS interaction in turn has been shown to reduce prostate cancer metastasis by blocking the expression of specific proteoglycans.^100^

**FIGURE 1:**
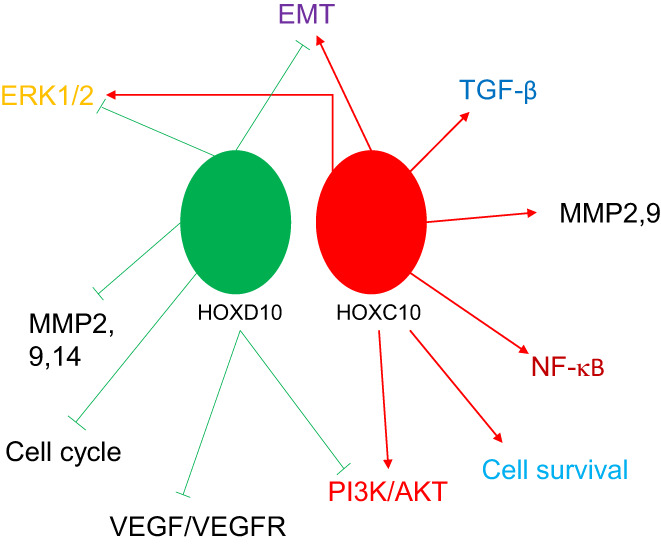
Target genes and pathways of HOXC10 and HOXD10. HOXC10 and its paralog HOXD10 have exclusively pro‐oncogenic and tumour suppressor functions, respectively. With the exception of the ERK1/2 and PI3K/AKT signalling pathways and genes involved in the epithelial to mesenchymal transition (EMT), there is little overlap in target genes. Arrows indicate activation of target genes and lines ending with a bar indicate repression [Color figure can be viewed at wileyonlinelibrary.com]

As discussed above, HOXD10 is exceptional amongst the HOX genes in acting solely as a tumour suppressor—there have been no functional studies of its mechanism published to our knowledge that do not report tumour suppressor activity, with the exception of HNSCC in which it seems to reduce cell invasion but increase cell proliferation.^124^ It is noteworthy though that its paralog in the C cluster, HOXC10, is one of a small group of HOX genes for which only pro‐oncogenic functions have been reported. Comparing the protein sequences of these two transcription factors using the BlastP application in PubMed (HOXC10—NP_059105 vs HOXD10—NP_002139) reveals that there is limited sequence identity outside of the homeodomain. This suggests that both proteins bind the same sequences in DNA, in which case the simplest explanation for their contrasting functions would be that they compete with each other to activate or repress target genes. However, comparing the target genes and pathways of HOXC10 and HOXD10 (Figure 1) suggests this may not be the case. Although there are a few common targets, mainly genes involved in the EMT^55^, ^66^, ^70^, ^71^, ^115^, ^117^ and in PI3K/Akt signalling,^58^, ^68^, ^70^, ^119^, ^120^ the majority of targets do not overlap. Hence HOXC10 activates the expression of the pro‐survival gene BCL2 and inhibits the pro‐apoptotic genes Bax and caspase 3,^71^, ^125^ while HOXD10 inhibits the expression of the anti‐apoptotic gene survivin.^111^ Likewise, HOXC10 activates genes involved in the NF‐κB pathway,^72^ while there is no evidence that HOXD10 has the opposite function. Thus, the opposing functions of HOXC10 and HOXD10 may not relate to a competition for binding to promoters and enhancers of the same target genes, but may instead represent largely independent mechanisms.

## 3 HOX GENES REGULATE DISCRETE FUNCTIONAL GROUPS OF GENES

**FIGURE 2:**
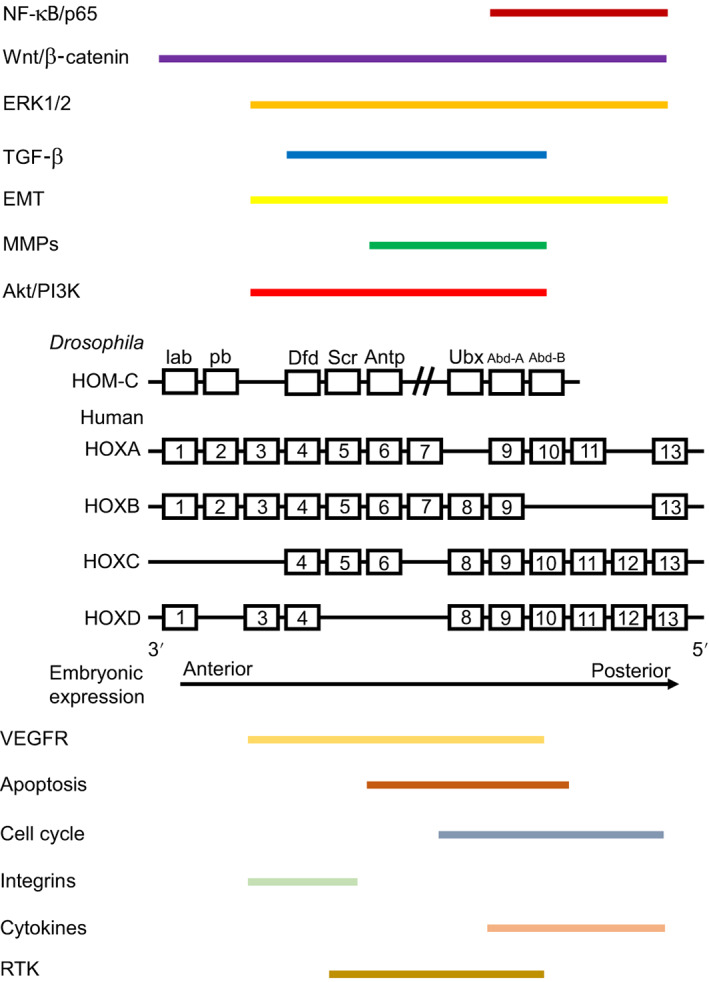
Mapping groups of functionally‐related target genes to HOX genes within each cluster. While some functional groups, for example, genes involved in wnt/β‐catenin signalling, are regulated by HOX genes throughout the clusters, others such as integrins are regulated by a relatively small group of genes [Color figure can be viewed at wileyonlinelibrary.com]

**FIGURE 3:**
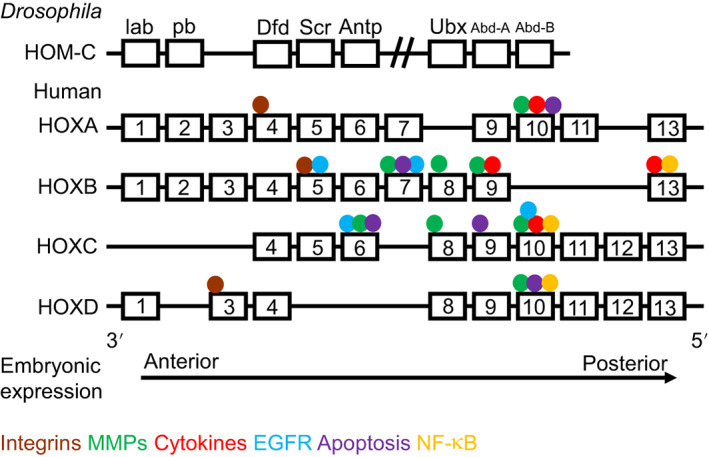
Mapping of HOX target genes that are involved in integrin function, cytokine/EGFR/NF‐κB signalling, or apoptosis, or which belong to the integrin or matrix metalloproteinase (MMP) families [Color figure can be viewed at wileyonlinelibrary.com]

From the currently available literature related to mechanistic studies of HOX genes in cancer, 13 distinct groups of targets can be identified that relate to specific functional subsets of genes (eg, EMT or apoptosis) or different signalling pathways (Figure 2). While some functions are mediated by HOX genes located throughout the clusters, others are restricted to smaller groups of paralog genes (Figure 3). For example, while HOX genes from across the whole cluster are involved in regulation of wnt/β‐catenin signalling,^22^, ^27^, ^49^, ^51^, ^83^, ^126^, ^127^, ^128^, ^129^, ^130^, ^131^, ^132^, ^133^, ^134^, ^135^, ^136^, ^137^, ^138^, ^139^, ^140^ for other HOX target pathways regulation is by a far more restricted group of genes. The later include the matrix metalloproteinases (MMPs), one of the most common HOX targets, which are only regulated by HOX genes from paralogous groups 6 to 10,^20^, ^37^, ^43^, ^51^, ^70^, ^71^, ^110^, ^111^, ^113^, ^115^, ^117^, ^118^, ^141^, ^142^, ^143^, ^144^, ^145^, ^146^ while integrins are regulated by paralog group genes 3 to 5,^140^, ^147^, ^148^, ^149^, ^150^, ^151^, ^152^ and targets related to the NF‐κB/p65 signalling pathway by groups 9 to 13.^63^, ^67^, ^72^, ^92^, ^94^, ^139^

**FIGURE 4:**
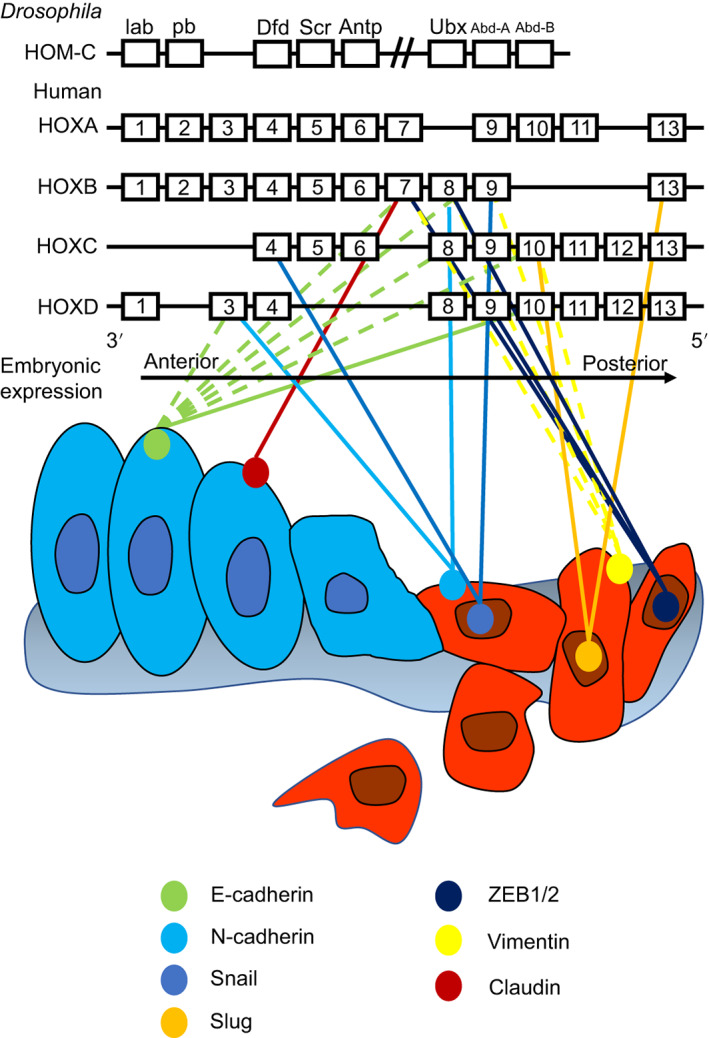
HOX target genes involved in the epithelial to mesenchymal transition (EMT). Solid lines indicate that the target gene is upregulated by the indicated HOX transcription factor while dashed lines denote repression. Vimentin, an intermediate filament protein characteristic of epithelial cells, is downregulated by HOXB9, HOXC10, HOXB8 and HOXB7. E‐cadherin, another transmembrane protein expressed in epithelial cells is also downregulated by multiple HOX genes in different cancers, with the exception of HOXD10 that activates its transcription, while its counterpart in mesenchymal cells, N‐cadherin, is upregulated by HOXD3 and HOXB8. Other upregulated HOX targets in EMT are the key transcription factors that promote this process—Snail (HOXB9 and HOXC4), Slug (HOXB13 and HOXC10) and ZEB1/2 (HOXB7, HOXB8 and HOXD9) [Color figure can be viewed at wileyonlinelibrary.com]

The most commonly reported targets of HOX proteins are genes involved in the EMT (Figure 4). These include vimentin, an intermediate filament protein characteristic of epithelial cells, which is downregulated by HOXB9 in ovarian cancer,^144^ HOXC10 in lung cancer,^70^ HOXB8 in colorectal cancer^51^, ^53^ and HOXB7 in breast cancer.^41^ E‐cadherin, another transmembrane protein expressed in epithelial cells, is also downregulated by multiple HOX genes in different cancers,^41^, ^53^, ^55^, ^70^, ^71^, ^148^, ^152^, ^153^, ^154^ while its counterpart in mesenchymal cells, N‐cadherin, is upregulated.^53^, ^55^, ^111^, ^148^, ^155^ Other HOX targets in EMT are the key transcription factors that promote this process—Snail, Slug and ZEB1/2. Notably, HOXB7 and HOXB8 are the most frequently linked to EMT—HOXB7 has been shown to regulate the expression of vimentin, claudin and E‐cadherin,^41^ while HOXB8 regulates E‐cadherin, N‐cadherin, vimentin^51^, ^53^ and ZEB1/2.^48^, ^53^ The second largest sets of HOX targets, the MMPs, are also involved in the EMT through their ability to remodel the extracellular matrix. MMP9 is upregulated by HOXB9 in ovarian^144^ and lung cancer,^145^ and by HOXC6 in gastric cancer,^141^ while MMP2 is upregulated by HOXB7^146^ and HOXC10^70^ in lung cancer, HOXB8 in colorectal cancer^51^ and HOXC10 in osteosarcoma.^71^ Notably, in cases where HOX genes seem to have a tumour suppressor function involving MMPs, the target of regulation is often MMP14 rather than MMP2 or MMP9. MMP14 is repressed by HOXD10 in pancreatic cancer,^111^ bladder cancer^115^ and glioma,^113^ while there is no evidence that it is activated by any of the other HOX proteins.

Other functional groupings of genes regulated by HOX proteins in cancer involve regulators of apoptosis, and in particular, caspase 3 that is repressed by HOXC13 in oesophageal cancer^125^ and HOXC10 in osteosarcoma,^71^ Bax that is upregulated by HOXC10 in osteosarcoma^71^ and the anti‐apoptotic gene BCL2 that is upregulated by HOXA10 in gastric cancer,^156^ HOXC10 in osteosarcoma^71^ and HOXC6 in HNSCC.^157^ In a similar finding as for the MMPs, the only reported tumour suppressor function for a HOX gene involving apoptosis acts on a different target; HOXD10 has been shown to block the expression of the anti‐apoptotic gene survivin in pancreatic cancer.^111^

The other functionally discrete sets of genes regulated by HOX genes in cancer are the integrins, cell cycle components, vascular endothelial growth factor (VEGF) and vascular endothelial growth factor receptor (VEGFR) (angiogenesis) and components of the TGF‐β, wnt/β‐catenin, receptor tyrosine kinase (RTK), Atk/PI3K, NF‐κB/p65 and cytokine (JAK/STAT) signalling pathways. Of these, the canonical wnt/β‐catenin pathway is the most common target of HOX regulation, being upregulated in lung cancer (HOXB5^134^, ^138^ and HOXB9^130^, ^132^), colorectal cancer (HOXB8),^51^ breast cancer (HOXD3),^140^ gastric cancer (HOXA13^158^ and HOXB5^129^), pancreatic cancer (HOXB5),^159^ osteosarcoma (HOXB8)^49^ and HNSCC (HOXB5).^131^ HOXB13 suppresses wnt/β‐catenin signalling in colon cancer, hence exhibiting a tumour suppressor function in this context, but does so through inhibiting transcription of the TCF4 gene (the product of which encodes a cofactor for β‐catenin).^103^

## 4 DIRECT HOX TARGETS

**FIGURE 5:**
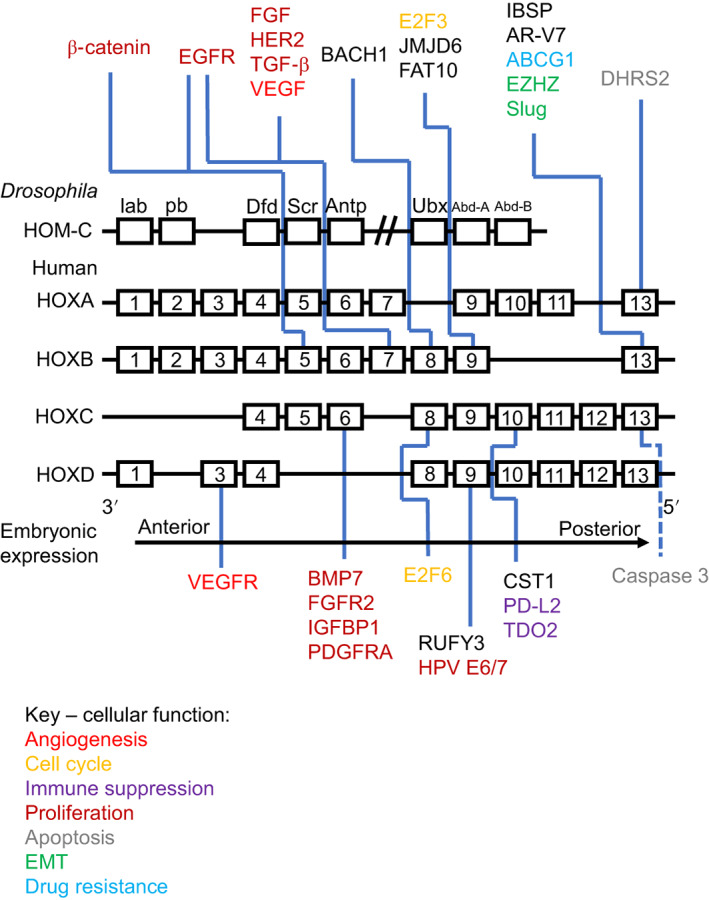
HOX target genes for which there is experimental evidence for HOX protein binding to the promoter/enhancer region, and a requirement for this binding for gene regulation. All the target genes are upregulated by HOX transcription factors, with the exception of caspase 3 which is repressed by HOXC13 [Color figure can be viewed at wileyonlinelibrary.com]

Amongst the publications that report mechanistic studies for the role of HOX genes in cancer, 28 provide experimental evidence for direct regulation of target genes (ie, HOX protein binding to the promoter/enhancer regions that is required to activate or repress transcription). Notably, all of these identify a pro‐oncogenic role for the HOX protein(s) being studied (Table 1). The directly regulated targets identified in these studies are summarised in Figure 5. With the exception of EGFR, which is upregulated by both HOXB5^160^ and HOXB7,^30^, ^31^ the HOX gene targets do not overlap but instead seem to be specific to individual HOX genes. However, there is a high degree of functional overlap mainly related to pro‐oncogenic pathways, so for example, in addition to EGFR, other RTKs that are upregulated include HER2 and VEGFR, by HOXB7^30^ and HOXD3,^161^ respectively.

A particularly notable finding is that HOXC10 can activate the transcription of two genes involved in immune suppression—Programmed Death Protein‐Ligand 2 (PD‐L2) and Tryptophan 2,3‐Dioxygenase (TDO2)—by binding directly to their promoters.^64^ PD‐L2 can bind to the PD‐1 receptor on T‐cells to block their activation,^162^ and TDO2 converts tryptophan to kynurenine, which also has an inhibitory effect on T‐cells.^163^ Elevated HOXC10 and TDO2 expression in glioma are both associated with immune suppression that supports tumour progression, indicating that HOX genes have functions in immunosuppression in addition to the other pro‐oncogenic roles described above.^64^

## 5 IMPLICATIONS FOR CANCER TREATMENT

Individual HOX genes have been identified as possible therapeutic targets in cancer based on their pro‐oncogenic function. However, there are a number of difficulties with this approach. One of these is the high degree of functional redundancy amongst HOX genes with respect to downstream target genes in cancer. Examples of these are discussed above but include genes involved in the EMT, where, with the possible exception of Claudin, target genes are regulated by two or more HOX proteins, and for E‐cadherin by at least six HOX proteins. One approach to overcoming this is to target multiple HOX proteins through their interaction with PBX using an inhibitory peptide (HXR9) that acts as a competitive antagonist by mimicking the conserved hexapeptide region in HOX paralogue group proteins 1 to 10 that mediates PBX binding.^164^ HXR9 has been shown to cause apoptosis in a range of cancers both in vitro and in vivo, including prostate,^8^ breast,^9^ lung,^10^ renal,^11^ ovarian,^165^ oral^12^ and oesophageal cancer,^166^ as well as mesothelioma,^14^ myeloma,^167^ melanoma^168^, ^169^ and acute myeloid leukaemia.^170^ A potential difficulty with this approach though is the apparent tumour suppressor function of some HOX genes, although, as described above, this is mainly reported from associative rather than mechanistic studies, with evidence from the latter strongly indicating a primarily pro‐oncogenic role for HOX genes.

An alternative would be a targeted approach using small‐molecule inhibitors based on the HOX expression profile of individual tumours. Previous work has focused on developing in vitro, cell‐based screens for drugs that target dysregulated HOX expression, and identified the histone deacetylase inhibitor Entinostat as a selective agent for leukaemia cells expressing high levels of HOX genes together with their TALE transcription factors.^171^ Effective, direct targeting of HOX transcription factors by small molecules has so far proved elusive, which is due in part to the lack of structural data for the majority of HOX proteins, and the inherent difficulty of targeting transcription factors that generally interact through large hydrophobic surfaces. However, the recent development of the AI‐driven AlphaFold model for predicting protein structures^172^ together with proteasome‐targeting technology may help facilitate this approach.^173^

## 6 CONCLUSIONS

The role of HOX genes in cancer has now been extensively studied, although this has resulted in a number of apparently contradictory findings, especially the conflicting roles of HOX genes as tumour suppressors vs promoters of oncogenesis. In order to better understand the mechanisms by which HOX genes act in cancer, we have considered only those studies that provide experimental evidence for HOX function, rather than associative studies restricted to the relationship between HOX expression and clinical outcomes. On this basis, it seems that the role of HOX genes in cancer is predominantly pro‐oncogenic, with the exception only of HOXD10. Furthermore, when grouped together by function, many target genes are regulated by only a few HOX proteins. This indicates that while HOX proteins are potential therapeutic targets in cancer, this may ultimately depend on an approach rooted in personalised medicine that adapts to the HOX expression profiles of individual tumours.

## CONFLICT OF INTEREST

The authors have no conflicts of interest to declare.
